# Automated Brain Tumor MRI Segmentation Using ARU-Net with Residual-Attention Modules

**DOI:** 10.3390/diagnostics15182326

**Published:** 2025-09-13

**Authors:** Erdal Özbay, Feyza Altunbey Özbay

**Affiliations:** 1 Department of Computer Engineering, Firat University, 23119 Elazig, Türkiye; 2 Department of Software Engineering, Firat University, 23119 Elazig, Türkiye; faltunbey@firat.edu.tr

**Keywords:** brain tumor, segmentation, attention Res-UNet, neural network, magnetic resonance imaging

## Abstract

**Background/Objectives:** Accurate segmentation of brain tumors in Magnetic Resonance Imaging (MRI) scans is critical for diagnosis and treatment planning due to their life-threatening nature. This study aims to develop a robust and automated method capable of precisely delineating heterogeneous tumor regions while improving segmentation accuracy and generalization. **Methods:** We propose Attention Res-UNet (ARU-Net), a novel Deep Learning (DL) architecture integrating residual connections, Adaptive Channel Attention (ACA), and Dimensional-space Triplet Attention (DTA) modules. The encoding module efficiently extracts and refines relevant feature information by applying ACA to the lower layers of convolutional and residual blocks. The DTA is fixed to the upper layers of the decoding module, decoupling channel weights to better extract and fuse multi-scale features, enhancing both performance and efficiency. Input MRI images are pre-processed using Contrast Limited Adaptive Histogram Equalization (CLAHE) for contrast enhancement, denoising filters, and Linear Kuwahara filtering to preserve edges while smoothing homogeneous regions. The network is trained using categorical cross-entropy loss with the Adam optimizer on the BTMRII dataset, and comparative experiments are conducted against baseline U-Net, DenseNet121, and Xception models. Performance is evaluated using accuracy, precision, recall, F1-score, Dice Similarity Coefficient (DSC), and Intersection over Union (IoU) metrics. **Results:** Baseline U-Net showed significant performance gains after adding residual connections and ACA modules, with DSC improving by approximately 3.3%, accuracy by 3.2%, IoU by 7.7%, and F1-score by 3.3%. ARU-Net further enhanced segmentation performance, achieving 98.3% accuracy, 98.1% DSC, 96.3% IoU, and a superior F1-score, representing additional improvements of 1.1–2.0% over the U-Net + Residual + ACA variant. Visualizations confirmed smoother boundaries and more precise tumor contours across all six tumor classes, highlighting ARU-Net’s ability to capture heterogeneous tumor structures and fine structural details more effectively than both baseline U-Net and other conventional DL models. **Conclusions:** ARU-Net, combined with an effective pre-processing strategy, provides a highly reliable and precise solution for automated brain tumor segmentation. Its improvements across multiple evaluation metrics over U-Net and other conventional models highlight its potential for clinical application and contribute novel insights to medical image analysis research.

## 1. Introduction

The growth of abnormal cells in the brain can lead to the formation of brain tumors, which are a major cause of morbidity and mortality worldwide. Gliomas, originating from glial cells, are broadly categorized into Higher Grade Gliomas (HGG) and Lower Grade Gliomas (LGG) [1]. HGGs grow rapidly with extensive cellular infiltration, whereas LGGs exhibit slower progression. Accurate and timely diagnosis is crucial for determining the appropriate treatment strategy. Non-invasive imaging techniques such as Magnetic Resonance Imaging (MRI), Positron Emission Tomography (PET), and Computed Tomography (CT) are widely used for tumor detection and monitoring. Among these, MRI has emerged as the preferred modality due to its superior ability to visualize soft tissue structures and provide high-resolution anatomical details [2].

Manual segmentation of brain tumors in MRI images requires expert radiological knowledge and is prone to inter- and intra-observer variability. Automatic segmentation methods offer a reliable alternative by enabling the quantitative assessment of the tumor size, location, morphology, and grade [3]. However, brain tumors, particularly gliomas, present significant challenges for segmentation due to their diffuse growth patterns, poor contrast, and intensity heterogeneity in MRI scans. Early approaches for MRI segmentation relied on classical image processing techniques, such as region growing, clustering-based methods, and watershed algorithms. Despite their utility, these methods are sensitive to noise and artifacts, limiting their overall effectiveness [4].

Recent advances in Deep Learning (DL) have demonstrated remarkable performance in brain tumor segmentation by learning complex hierarchical features from MRI data [5]. Convolutional Neural Networks (CNNs) and Fully Convolutional Networks (FCNs), including models such as U-Net [6], DenseNet121 [7], Xception [8], Deep Neural Networks (DNNs) [9], and their variants, have been successfully applied to this task, showing substantial improvements over traditional techniques. Building upon these advances, attention mechanisms such as Adaptive Channel Attention (ACA) [10] and Triplet Attention [11] have been employed to enhance feature representation and multi-scale contextual understanding. Inspired by these developments, this study proposes Attention Res-UNet (ARU-Net), an improved U-Net-based [12] architecture incorporating residual connections, ACA, and Dimensional-space Triplet Attention (DTA) [13] modules. Accurate tumor identification and segmentation are made possible by the network’s design, which maximizes local cross-channel interactions while reducing the model complexity. Furthermore, the study emphasizes the importance of pre-processing steps, such as Contrast Limited Adaptive Histogram Equalization (CLAHE), denoising, and Linear Kuwahara filtering, to enhance image quality, improve feature extraction, and facilitate more accurate segmentation [14]. The main contributions of this paper are summarized as follows:

- Pre-processing effectiveness: We demonstrate that applying CLAHE, denoising, and Linear Kuwahara filtering significantly enhances the MRI image quality and directly improves the segmentation performance across multiple tumor classes.
- Novel ARU-Net architecture: We propose a U-shaped deep neural network that integrates residual connections with dual attention modules to better capture both channel and spatial dependencies.
- Adaptive Channel Attention (ACA) in the encoder: Applied to the lower convolutional and residual layers, ACA improves the feature refinement and strengthens the representation learning.
- Dimensional-space Triplet Attention (DTA) in the decoder: Applied to the upper convolutional layers, DTA enables more effective extraction and fusion of multi-scale features, leading to smoother and more accurate tumor boundaries.
- Comprehensive evaluation: Experimental results on the BTMRII dataset demonstrate that ARU-Net consistently outperforms U-Net, DenseNet121, and Xception, achieving superior quantitative metrics and qualitative segmentation quality.

The remainder of the paper is organized as follows: Section 2 reviews the related DL approaches for brain tumor segmentation. Section 3 details the methodology, including the network architecture and component descriptions. Section 4 presents the experimental results, ablation studies, and comparative analyses. Section 5 discusses the experimental results and explains the limitations of the proposed method. Finally, Section 6 concludes the study and discusses future research directions.

## 2. Related Works

Brain tumor segmentation has long been a critical research area in medical image analysis due to its importance in clinical decision-making. Early studies were dominated by classical image processing techniques such as region-growing [15], clustering-based approaches [16], and watershed algorithms [17]. However, these methods were highly sensitive to noise, low contrast, and heterogeneous tumor appearance in MRI scans [18], limiting their accuracy, particularly for complex glioma structures [19].

With the advent of deep learning, CNN-based methods have rapidly replaced traditional approaches, achieving superior segmentation performance [20]. Several architectures have extended the U-Net by incorporating residual learning and advanced modules. Examples include cascaded residual multi-scale convolutions [21], multi-scale contextual attention [22], multimodal spatial-boundary integration [23], and deep residual encoders within U-Net [24]. Other works, such as ResUNet-a [25] and EffUNet++ [26], enhanced feature fusion and skip connections, while hybrid models like 3DUV-NetR+ [27] combined U-Net, V-Net, and Transformer encoders for richer contextual representation. These CNN-based approaches demonstrate the potential of hierarchical feature extraction but often face challenges in balancing computational complexity and segmentation detail.

Attention-based CNN extensions have also shown strong performance. Khorasani et al. embedded pre-trained backbones into U-Net for glioma subregion segmentation [28], while DTASUnet [29] applied dual-transformer attention to capture 3D volumetric context. Multi-task frameworks, such as the ensemble model by Wen et al. [30], combined segmentation with tumor grading, and modality-specific studies [31] highlighted the critical impact of input MRI sequences on accuracy.

More recently, Transformer and Mamba-based designs have gained attention. The SLCA-UNet [32] incorporated spatial and local channel attention into the U-Net, while the Adaptive Cascaded Transformer U-Net [33] employed cascaded transformer blocks for long-range dependency modeling. Wang et al. proposed S^3^-Mamba [34], optimized for small tumor segmentation, and Zhang et al. introduced the Edge-Interaction Mamba Network [35] for refined boundary delineation. These works emphasize global context modeling and edge-sensitive learning, offering strong performance in heterogeneous tumor cases.

Overall, CNNs and FCNs have laid the foundation for modern brain tumor segmentation [5,6,7,8,36], while recent efforts have focused on integrating attention mechanisms [37,38,39], residual learning [40], and hybrid architectures [41]. Despite these advances, multi-scale tumor morphology and fine-grained boundaries remain challenging [42]. Motivated by these limitations, the proposed ARU-Net integrates residual connections with dual attention mechanisms and enhanced pre-processing to achieve robust segmentation performance on the BTMRII dataset.

## 3. Materials and Methods

### 3.1. Dataset

**Figure 1:**
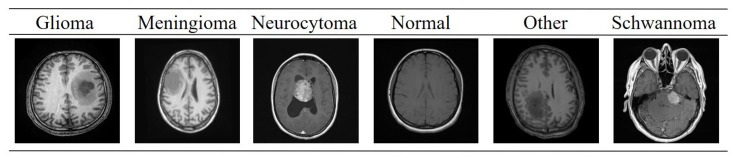
Sample images from each of the six classes in the BTMRII dataset.

The brain tumor MRI images used in this study were obtained from the BTMRII dataset, shared on the public platform Kaggle [43]. This dataset is an open collection widely referenced in research and comparative experiments. The original BTMRII dataset contains a total of 4448 real MRI images. The images were acquired in the axial plane and include T1, T1-enhanced (T1C+), and T2 modalities with different sequence weights. The original resolution and bit depth of the images may vary within the dataset. Therefore, firstly, all images in the study were rescaled to a uniform network input size of 256 × 256. BTMRII is primarily divided into six classes: Glioma, Meningioma, Neurocytoma, Normal, Other, and Schwannoma. The ‘Normal’ class is divided into two classes: T1 and T2, while the other classes are further divided into three classes: T1, T1C+, and T2, dividing the dataset into a total of 17 different classes. Cataloging is organized with a folder for each class. The set covers a variety of primary brain tumor histologies and some neurological lesion subtypes (example classes: astrocytoma, ependymoma, ganglioglioma, glioblastoma, oligodendroglioma, medulloblastoma, meningioma, pituitary/pituitary adenoma, schwannoma, germinoma, etc.). In this study, the T1, T1C+, and T2 modalities of the six main classes of BTMRII were combined. Thus, 1317 brain MRI images belonging to the Glioma class, 1299 to the Meningioma class, 542 to the Neurocytoma class, 563 to the Normal class, 257 to the Other class, and 470 to the Schwannoma class were used, all in 8-bit and jpeg format. The ground truth annotations were directly obtained from the BTMRII dataset, in which expert radiologists manually delineated the tumor regions on MRI scans. The annotation protocol followed a multi-class segmentation scheme, where each image was labeled with one of the 5 basic predefined tumor categories. For each class, the entire tumor region was annotated as a single mask, rather than subdividing into intra-tumoral components such as necrosis, edema, or enhancing tumor core. Thus, each mask corresponds to a binary segmentation map distinguishing tumor versus non-tumor regions for the respective tumor class. Example images from each of the six classes of the BTMRII dataset are shown in Figure 1.

### 3.2. Pre-Processing

In this study, a three-stage pre-processing strategy was employed to improve the success of the segmentation model in MRI-based brain tumor images. First, CLAHE enhances the visibility of tumor structures by locally increasing the contrast between the tumors and surrounding tissues. However, this process also carries the risk of introducing noise; therefore, a powerful denoising method such as non-local means denoising is then applied to suppress random noise in the image. In the final stage, edge-preserving smoothing is performed with a Linear Kuwahara filter to preserve the edge details while smoothing the overall textural image. This sequence both corrects for density artifacts and helps compensate for noise that may occur after CLAHE application. The CLAHE, Denoising, and Kuwahara filter sequence is the ideal approach for suppressing post-contrast noise and preserving edges. This three-stage approach aimed to increase the segmentation performance of the model while preserving the anatomical accuracy of the tumor. Low-frequency illumination/bias (intensity inhomogeneity) in MRI images, resulting from coil and magnetic field imbalances, can affect segmentation and contrast processing. Therefore, algorithms applied in the pre-processing steps aim to address this imbalance [44].

CLAHE enhances local contrast, facilitating the separation of tumor/edema regions from their surroundings. For this purpose, each MR image is divided into *I*(*x*, *y*) tiles. Histogram equalization is performed for each tile; histogram bins are limited by a clip limit to prevent excessive contrast enhancement; transitions between blocks are smoothed using bilinear interpolation. The mathematical formula is given in Equation (1) [45]:

(1) $$I^{\prime }=round\left(\frac{L-1}{MN}\sum_{k=0}^{i}h(k)\right)$$

Here, *L* is the number of gray levels, *h*(*k*) is the histogram frequency, and *MN* is the number of pixels within the tile. Denoising preserves structural similarities while reducing random noise [46]. In particular, the possibility of increased noise after CLAHE is compensated for. For this, a weighted average of similar patches in the surrounding area was taken for each pixel in the brain MRI images. The mathematical formula for the denoising process is given in Equation (2):

(2) $$\hat{I}\left(x\right)=\sum_{y\epsilon \Omega }w\left(x,y\right)\;I\left(y\right),\;w\left(x,y\right)\propto exp\left(-\frac{{\left\|P\left(x\right)-P\left(y\right)\right\|}_{2}^{2}}{h^{2}}\right)$$

Here, *P*(⋅) are the patch vectors, and *h* is the smoothing parameter in the range of 0.8–1.2.

The Linear Kuwahara filter was used to smooth the brain MRI images while preserving their edges [47]. Therefore, the smoothing was performed by subtracting the mean of the low-variance subregion. Each image window *W* is divided into four sub-regions; for each region, the mean *μk* and variance σ are calculated as shown in Equation (3):

(3) $$\mu_{k}=\frac{1}{\left|R_{k}\right|}\sum_{(i,j)\epsilon R_{k}}I\left(i,j\right),\;\sigma_{k}^{2}=\frac{1}{\left|R_{k}\right|}{\sum_{(i,j)\epsilon R_{k}}(I\left(i,j\right)-\mu_{k})}^{2}$$

The average of the region with the lowest variance is selected as the output pixel, as given in Equation (4):

(4) $${I^{\prime }(x,y)=\mu }_{k^{*}},\;k^{*}=arg\underset{k}{\min}\sigma_{k}^{2}$$

**Figure 2:**
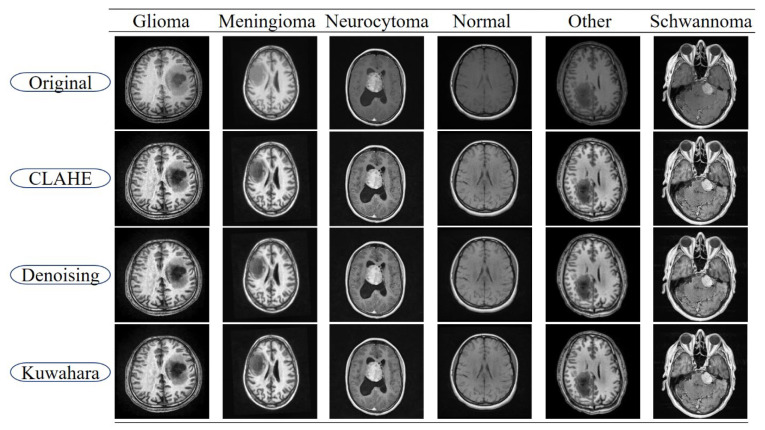
Sample images of each pre-processed stage from the BTMRII dataset.

In the linear variants, weighted sums of subregion averages were computed, yielding more stable outcomes. A 5 × 5 square window was employed. Consequently, the linear variants demonstrated greater robustness against block selection instability. Figure 2 illustrates sample images from each pre-processing stage of the BTMRII dataset.

### 3.3. General Framework

**Figure 3:**
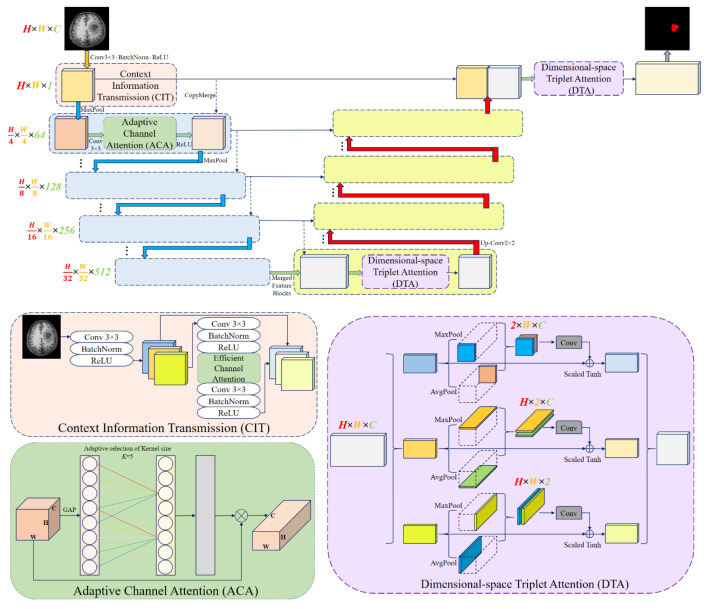
General framework of the implemented ARU-Net network with three branches.

In this study, the ARU-Net architecture was adopted as the baseline to achieve enhanced segmentation performance for brain tumor detection. The developed ARU-Net model consists of three main components: Context Information Transmission (CIT) [48], ACA, and DTA. As illustrated in Figure 3, which depicts the general structure of the improved ARU-Net with three branches, the CIT module aligns and transfers the features extracted before dimensionality reduction with those obtained after dimensionality reduction. This ensures that important structural information is preserved at different resolution levels. The ACA module emphasizes prominent channels after dimensionality reduction and automatically adjusts the weight distribution across different channel features. Dimensional-space attention models the relationships between the channel and spatial dimensions (height and width), enabling the extraction of global contextual information [49].

The input image has dimensions *H* × *W* × *C*. It is processed through convolutional layers (convolution, normalization, and activation) and max-pooling layers, progressively reducing the feature map’s number of channels and spatial resolution. The output dimensions after convolution are *H*′ × *W*′ × *C*′, where *H*′, *W*′, and *C*′ depend on the filter size *K* × *K*, stride *S*, and padding *P* parameters. After pooling, the dimensions are updated to *H*′′ × *W*′′ × *C*′′ [49]. Equation (5) provides the weights in the channel attention mechanism:

(5) $$\omega =tanh(\alpha \cdot (U_{a},U_{b})(g(z)))$$

Here, $g(z)=\frac{1}{W\cdot H}\sum_{i=1}^{H}\sum_{j=1}^{W}z_{ij}$, which denotes the Global Average Pooling (GAP) [50], while tanh (α·x) represents the scaled hyperbolic tangent activation. The parameter α is a scaling factor determined empirically. The dimensions of the weight matrices $U_{a}$ and $U_{b}$ are chosen as $C\times \left(\frac{C}{r}\right)$ and $\left(\frac{C}{r}\right)\times C$, respectively. While the feature maps’ size gradually decreases as the encoder blocks increase, the decoder blocks perform the reverse operation to restore the original dimensions. A channel attention layer, a skip connection, and a downsampling unit make up each encoder module. On the decoder side, transposed convolution, the dimensional-space attention module, and skip connections are used for feature fusion [49]. Finally, a single convolution layer produces the final segmentation output.

#### 3.3.1. Context Information Transmission (CIT)

**Figure 4:**
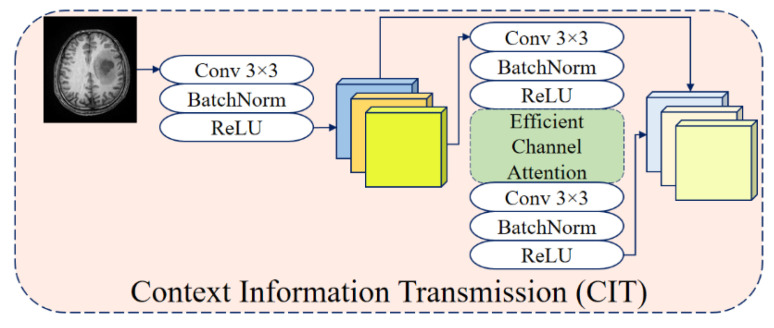
The procedure of the basic CIT module.

The CIT module begins by processing the input image with a 3 × 3 convolution, batch normalization, and ReLU activation [51]. The procedure of the basic CIT module is shown in Figure 4, and the mathematical formulation is given in Equation (6):

(6) $$Q_{1}=ReLU(BN({Conv}_{3\times 3}(I)))$$

Here, *Q*_1_ denotes the primary feature map extracted from the input, preserving essential information. This map is passed through a fully connected layer and forwarded to the encoder. Subsequently, *Q*_1_ undergoes dimensionality reduction via max-pooling to produce *Q*_2_. *Q*_2_ is processed by the scaled hyperbolic tangent activation and the ACA mechanism. The output of ACA is re-activated to obtain *Q*_3_. In the final step, *Q*_1_ and *Q*_3_ are summed as *R*_1_ = *Q*_1_ + *Q*_3_, which serves as the input feature for subsequent layers. The ACA learns the inter-channel relationships and optimizes the weight of each channel. After obtaining global contextual information using GAP, a lightweight attention mechanism enhances the feature representation. This ensures stable gradient propagation even as the model depth increases, resulting in a more efficient learning process.

#### 3.3.2. Adaptive Channel Attention (ACA)

**Figure 5:**
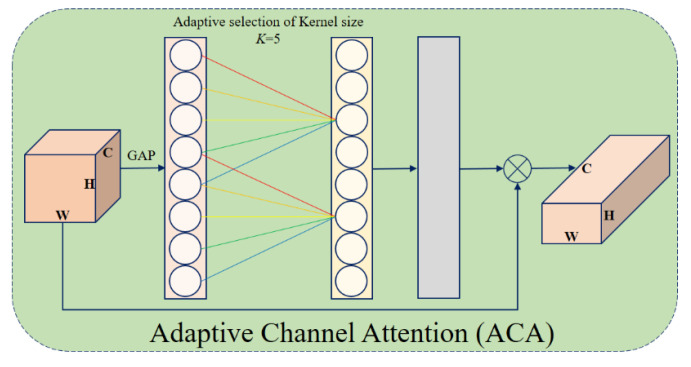
The procedure of the basic ACA module.

The ACA module employs a 1D convolution-based structure to capture inter-channel dependencies between neighboring channels [52]. The procedure of the basic ACA module is shown in Figure 5, and its mathematical formulation is given in Equation (7):

(7) $$\omega =tanh(\alpha \cdot C_{1}D_{k}(y))$$

Here, *k* denotes kernel size, and *C*_1_*D*_k_ is a 1D convolution. The value of *k* is adaptively determined based on the number of channels (*C*). This relationship is approximated by an exponential function, and with parameters *γ* = 2 and *β* = 1, it is computed as in Equation (8):

(8) $$C=\phi (k),\;k\approx {\left|\frac{{log}_{2}(C)}{\gamma }+\frac{\beta }{\gamma }\right|}_{odd}$$

This adaptive process enables the ARU-Net model, which was created for the purpose of detecting brain tumors, to highlight significant channels in multi-channel data and capture tumor traits at various sizes.

#### 3.3.3. Dimensional-Space Triplet Attention (DTA)

**Figure 6:**
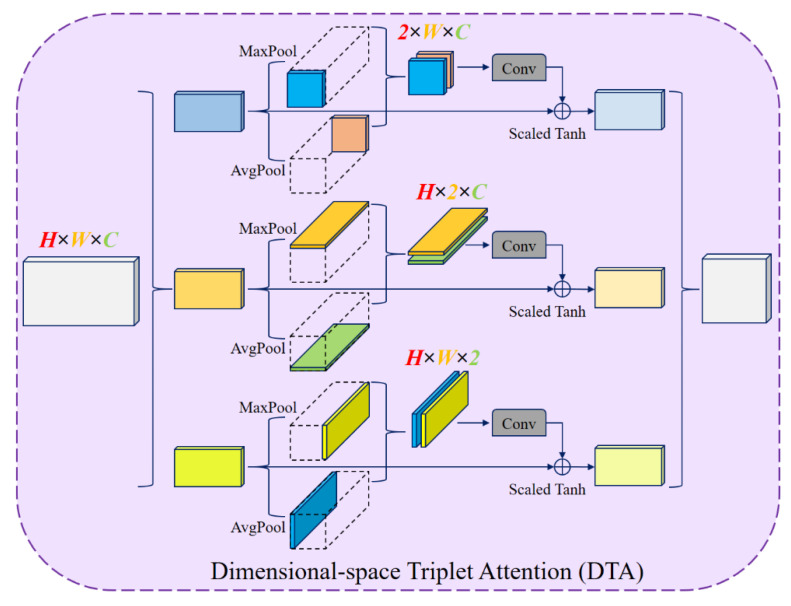
The procedure of the basic DTA module.

During feature fusion on the decoder side, an enhanced dimensional-space attention module is employed [53]. The procedure of the basic DTA module is given in Figure 6. This module captures interactions between channel and spatial dimensions through three parallel branches. The input tensor *x* ∈ ℝ (*H* × *W* × *C*) is processed in each branch across different dimension pairs using pooling, convolution, and scaled tanh activation. For instance, height and channel dimensions interact in the first branch, the width dimension is reduced to 1 through both max and average pooling, and the resulting two feature maps of size 1 × *W* × *C* are concatenated to form a 2 × *W* × *C* feature map. Convolution and activation are then applied. Similarly, the second branch captures the width–channel relationships, while the third branch captures the height–width relationships [54]. The outputs of the three branches are combined as given in Equation (9):

(9) $$y=\frac{1}{3}\cdot ({\hat{x}}_{1}\cdot \omega_{1}+{\hat{x}}_{2}\cdot \omega_{2}+{\hat{x}}_{3}\cdot \omega_{3})$$

Here, *ω*_1_, *ω*_2_, and *ω*_3_ are the cross-dimensional attention weights, computed using the scaled tanh activation as in Equation (10):

(10) $$\omega_{a}=\tanh\left(\alpha \cdot \psi_{a}({{\hat{x}}_{a}}^{*})\right)$$

where $\psi_{a}$ denotes the standard 2D convolution, and *a* ∈ {1, 2, 3} is the branch index.

To assess the segmentation performance of the proposed ARU-Net and baseline models, we employed several widely used quantitative evaluation metrics, including accuracy, precision, recall, F1-score, DSC, and IoU. These metrics provide complementary perspectives on the classification correctness, overlap quality, and boundary precision, and their mathematical definitions are given in the Section 4, alongside the reported experimental outcomes.

## 4. Experimental Results

In order to evaluate the effectiveness of the proposed model, a series of experiments were conducted under a controlled computational environment. The training and testing processes were implemented in Python 3.10 using the PyTorch 2.1.0 framework, running on a workstation equipped with an Intel Core i7 4.70 GHz processor, an NVIDIA RTX 4060Ti GPU, and 32 GB of RAM. During training, a batch size of six samples was employed to stabilize the gradient updates, while the Adam optimizer was adopted to achieve efficient convergence. In the pre-processing stage, all images from the BTMRII dataset were resized to ensure uniformity across the samples. To guarantee a fair evaluation, the dataset was randomly divided into three independent subsets: 60% for training, 20% for validation, and 20% for testing, following a 6:2:2 ratio. The results obtained from this setup are presented and analyzed in the subsequent subsections, with comparisons drawn against the related works in the literature.

In addition to the proposed framework, several well-established DL architectures were also trained for comparative analysis. DenseNet121 and Xception models were implemented using the TensorFlow/Keras 2.15 environment. The input resolution was set to 224 × 224 for DenseNet121 and 299 × 299 for Xception, respectively. Both models employed the Adamax optimizer (learning rate = 0.001), along with an early stopping strategy (patience = 10, based on validation accuracy) to prevent overfitting. The best-performing checkpoints were stored during training for subsequent evaluation. For the U-Net configuration, the network was structured as a segmentation-based classifier, where the encoder backbone was initialized with EfficientNetB0. The input resolution was set to 224 × 224, and training was performed using the Adamax optimizer (learning rate = 1 × 10^−4^). To enhance the training stability, the encoder weights were frozen in the initial stages before fine-tuning. As with the classification models, the training and validation losses and accuracies were monitored, while the performance was further evaluated using classification reports, confusion matrices, and multi-class ROC curves generated on the test set. Both the best-performing models (based on validation metrics) and the final trained models were preserved. The proposed ARU-Net architecture was developed and trained within the PyTorch/timm framework. For this model, an input resolution of 256 × 256 was selected, and the Adam optimizer (learning rate = 1 × 10^−4^) was used during training. Unlike the baseline models, the checkpoint selection criterion for ARU-Net was based on the validation weighted F1-score, enabling a more balanced performance assessment across multiple tumor classes. For the final evaluation, 20% of the BTMRII dataset was reserved as the independent test set, ensuring that no data leakage occurred between training and evaluation. This subset contained representative samples from all classes, thereby enabling a balanced assessment of classification and segmentation performance. Specifically, the test set consisted of 258 Glioma, 260 Meningioma, 108 Neurocytoma, 113 Normal, 51 Other, and 94 Schwannoma classes. The inclusion of multiple tumor subtypes in the evaluation phase ensured that the models were validated under diverse clinical scenarios, reflecting both common and relatively rare brain tumor categories.

In this study, DenseNet121, Xception, U-Net, and the proposed ARU-Net were selected for evaluation based on their complementary strengths and established performance in medical image analysis. DenseNet121, with its densely connected convolutional blocks, enables efficient feature reuse and mitigates vanishing gradient problems, making it a strong candidate for extracting discriminative features from MRI data. Xception, on the other hand, leverages depthwise separable convolutions to capture fine-grained spatial features while maintaining computational efficiency, which is particularly advantageous for high-resolution medical imaging tasks. For segmentation-oriented classification, U-Net was incorporated due to its widespread success in biomedical image segmentation, where the encoder–decoder structure and skip connections allow precise localization of tumor regions. To further advance this line of research, the proposed ARU-Net model was developed, integrating architectural refinements and an optimized training strategy to better handle class imbalance and heterogeneous tumor morphology. The inclusion of these models provided both baseline comparisons with well-established DL architectures and a platform to demonstrate the added value of the proposed ARU-Net in brain tumor classification and segmentation.

To address the class imbalance within the BTMRII dataset, we employed two complementary strategies. First, we utilized a class-weighted categorical cross-entropy loss function, where higher weights were assigned to underrepresented classes. This adjustment penalized misclassification of rare tumor types more strongly, guiding the model toward improved balance across categories. Second, a balanced mini-batch sampling procedure was adopted to ensure that each training batch contained approximately equal representation from both majority and minority tumor classes. This reduced the risk of biased learning toward dominant categories. During the training phase, categorical cross-entropy loss was employed as the objective function for all classification-based models, including DenseNet121, Xception, U-Net, and the proposed ARU-Net. Cross-entropy is widely recognized as a suitable loss function for multi-class classification problems, as it measures the dissimilarity between the true label distribution and the predicted probability distribution [55]. Penalizing incorrect predictions more strongly enables stable convergence and effective optimization when combined with gradient-based learning. For the proposed ARU-Net, enhanced from the U-Net architecture, which was adapted as a segmentation-oriented classifier in this study, cross-entropy loss was also adopted to maintain consistency in evaluation and to ensure comparability across models. Its mathematical formula is given in Equation (11):

(11) $$L=-\frac{1}{N}\sum_{i=1}^{N}\sum_{c=1}^{C}y_{i,c}\log{(\hat{y}}_{i,c})$$

Here, the total number of samples is defined by *N*, *C* defines the total number of classes, *y* defines the true label, and the probability that it is predicted by the model. To comprehensively assess the segmentation performance of the proposed ARU-Net model, multiple evaluation metrics were employed. These include the accuracy, which is given in Equation (12); the precision, which is given in Equation (13), the recall, which is given in Equation (14); the F1-score, which is given in Equation (15) [56]; the IoU, which is given in Equation (16); and the DSC, which is given in Equation (17) [57]. Each of these metrics provides complementary insights into the effectiveness of the segmentation by quantifying different aspects of the overlap between the predicted and ground truth regions. Their mathematical definitions are given as follows:

(12) $$Accuracy=\frac{TP+TN}{TP+TN+FP+FN}$$

(13) $$Precision=\frac{TP}{TP+FP}$$

(14) $$Recall=\frac{TP}{TP+FN}\;$$

(15) $$F1-score=2\cdot \frac{Precision\cdot Precision}{Precision+Precision}\;$$

(16) $$IoU=\frac{TP}{TP+FP+FN}$$

(17) $$DSC=\frac{2TP}{2TP+FP+FN}$$

Here, *TP* (True Positive), *TN* (True Negative), *FP* (False Positive), and *FN* (False Negative) denote the fundamental components of the confusion matrix. While the IoU and DSC focus on evaluating the overlap between the predicted and true tumor regions, the precision and recall quantify the model’s ability to correctly detect positive samples without excessive false alarms. The accuracy gives an overall measure of the classification correctness, whereas the F1-score balances the precision and recall, making it especially useful in the presence of class imbalance.

### Performance Analysis

To systematically compare the effectiveness of different DL approaches in brain tumor segmentation, a series of experiments were conducted using the BTMRII dataset, and the corresponding results are organized into comprehensive tables and visualizations. Table 1 presents a detailed summary of eight evaluation metrics including the accuracy, precision, recall, F1-score, IoU, DSC, GFlops, and the number of parameters (Param) obtained from four state-of-the-art DL-based methods. These metrics collectively provide both computational and predictive performance insights, enabling a thorough assessment of each model. To visually illustrate the performance differences among the evaluated methods, representative segmentation results are provided, demonstrating the models’ ability to delineate tumor boundaries accurately. Additionally, to validate the quality and reliability of the segmentation outcomes, various diagnostic tools were employed, including confusion matrices, accuracy and loss curves, and multiclass ROC curves, which further highlight the precision and robustness of the segmented regions across different tumor categories. Furthermore, to assess the effectiveness of the pre-processing pipeline applied to the BTMRII dataset, Table 1 also includes comparisons between the segmentation results obtained from the original images and those pre-processed through CLAHE, denoising, and Linear Kuwahara filtering. This comparative analysis provides a clear demonstration of how the pre-processing steps enhance the model performance and improve the quality of tumor delineation.

In this study, the performance of four DL models, DenseNet121, Xception, U-Net, and the proposed ARU-Net, was systematically evaluated on the BTMRII dataset using both original (raw) and pre-processed MRI images. Table 1 summarizes the comparative performance metrics. The experimental results indicate a consistent improvement in segmentation and classification performance when pre-processing techniques (CLAHE, denoising, and Linear Kuwahara filtering) were applied. For instance, DenseNet121 achieved an accuracy increase from 77.5% on the raw images to 82.2% on the pre-processed images, accompanied by improvements in the precision (82.0–86.3%) and F1-score (78.4–82.4%). Similarly, Xception showed a gain in accuracy from 81.0% to 85.3%, and U-Net improved from 90.0% to 93.9%. The ARU-Net model, which already demonstrated the highest baseline performance, benefited further from pre-processing, achieving 98.3% accuracy compared to 96.0% on the raw images. These results clearly demonstrate that appropriate pre-processing enhances the feature contrast, reduces noise, and preserves structural details, which collectively contribute to more accurate segmentation and classification outcomes.

U-Net also performed strongly, achieving 93.9% accuracy on pre-processed images, which underscores the advantage of encoder–decoder architectures for pixel-wise segmentation tasks. DenseNet121 and Xception, while primarily designed for image-level classification, achieved reasonable performance improvements with pre-processing but were outperformed by U-Net and ARU-Net in metrics sensitive to spatial segmentation quality (IoU and DSC).

To evaluate the computational efficiency of each network, the training time per epoch was measured on an NVIDIA RTX 4060Ti GPU using a batch size of 8. This metric complements the reported accuracy and model complexity (GFlops and parameter count) by providing practical insights into the relative training speed of each architecture.

**Figure 7:**
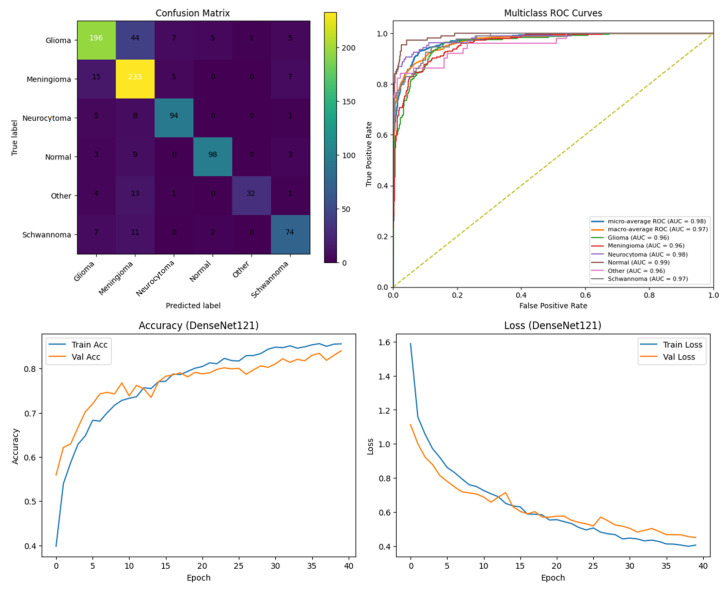
Performance results of the DenseNet121 model.

**Figure 8:**
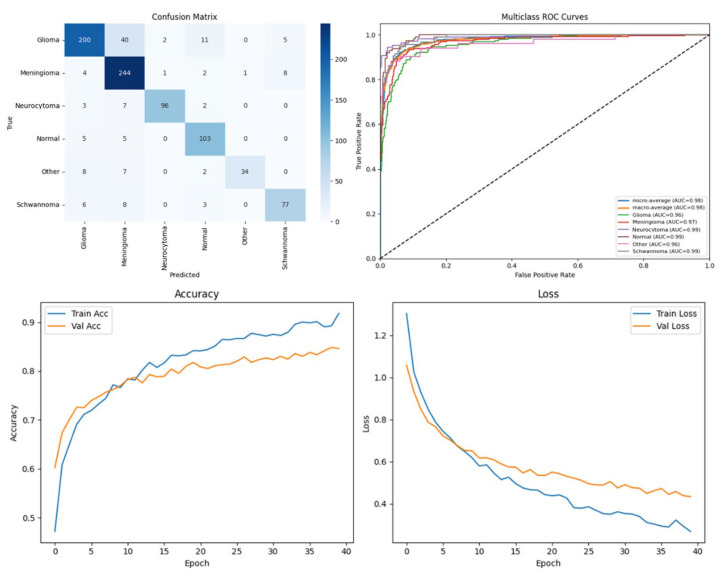
Performance results of the Xception model.

**Figure 9:**
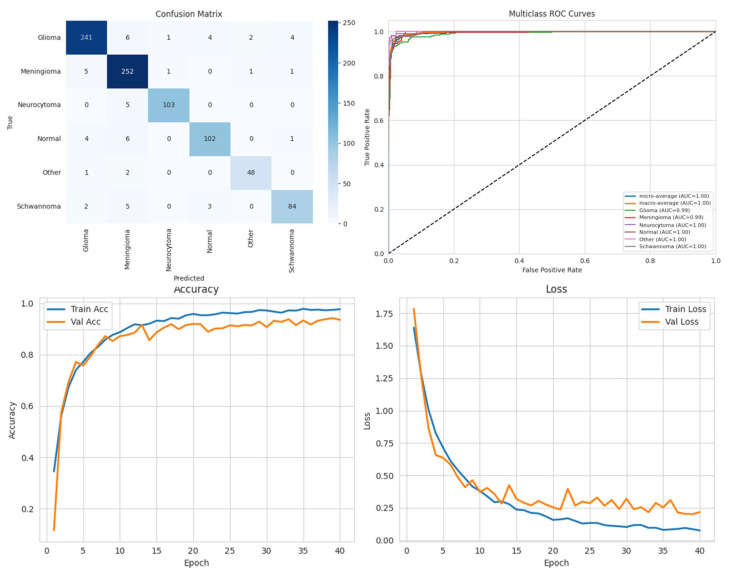
Performance results of the U-Net model.

**Figure 10:**
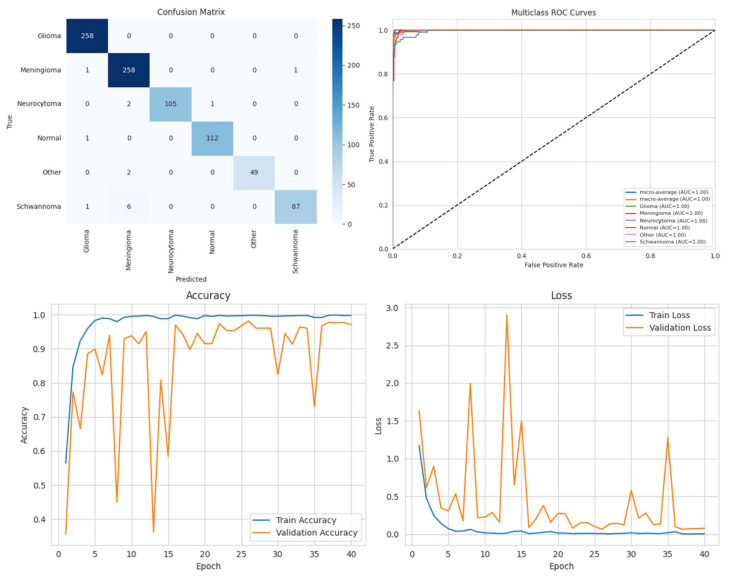
Performance results of the proposed ARU-Net model.

**Figure 11:**
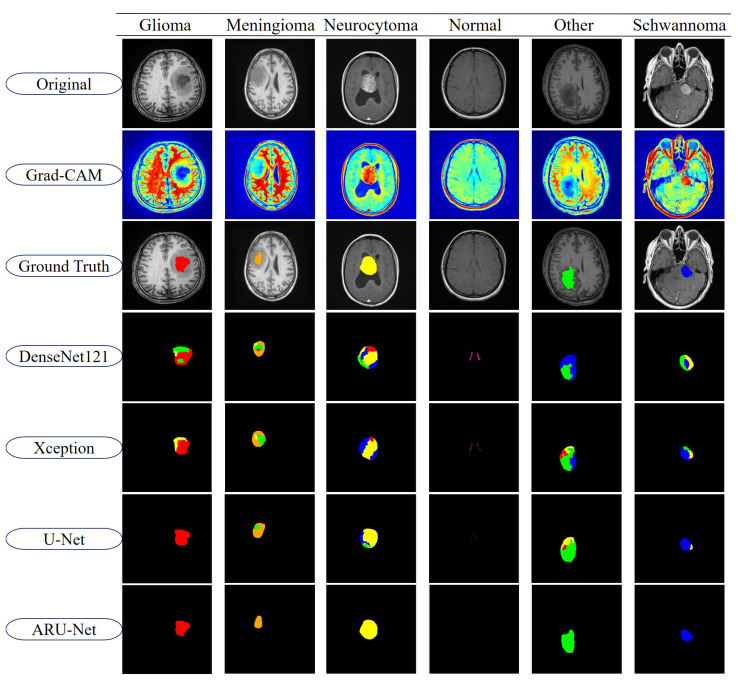
Segmentation results of the models for BRMRII dataset.

Among the four models, ARU-Net consistently outperformed the others across all metrics and dataset types. On the pre-processed images, ARU-Net achieved the highest precision (99.0%), recall (95.7%), F1-score (98.1%), IoU (96.3%), and DSC (98.1%), while maintaining moderate computational complexity (788.4 GFlops) and a manageable number of parameters (30.45 M). This demonstrates the efficacy of its attention-based architecture and EfficientNetB0 encoder in capturing multi-scale contextual information. Confusion matrices, accuracy and loss curves, and multiclass ROC curves of the pre-processed experiment results of the BTMRII dataset for four different models are shown in Figure 7, Figure 8, Figure 9, Figure 10 and Figure 11.

Comparing the models on the raw versus pre-processed images highlights the importance of data enhancement strategies. All models exhibited lower performance on the raw images, particularly in IoU and DSC metrics, which are directly related to the segmentation quality. For example, DenseNet121’s IoU decreased from 70.3% (pre-processed) to 65.0% (raw), and ARU-Net’s DSC decreased from 98.1% to 95.0%. These differences suggest that pre-processing not only improves the classification accuracy but also enables models to better delineate tumor boundaries, which is critical for clinical applicability.

To mitigate the potential overfitting observed in the training curves spanning 0 to 40 epochs (plotted in increments of 5), early stopping was applied based on validation loss with a patience of 10 epochs. Additionally, the input images were normalized, and the pre-processing steps including CLAHE, denoising, and Linear Kuwahara filtering were used to enhance the feature quality. A mini batch size of eight provided balanced gradient stability with model generalization.

The comparative analysis also includes the computational efficiency. While U-Net has higher GFlops (796.5) due to its encoder–decoder design, ARU-Net achieves superior performance with lower computational cost (788.4 GFlops), demonstrating a favorable balance between accuracy and efficiency. DenseNet121 and Xception, although deeper and parameter-heavy, did not achieve comparable segmentation metrics, indicating that the model architecture and suitability for segmentation tasks are as important as the model depth and parameter count.

Overall, these results emphasize three key points: (i) the application of pre-processing significantly enhances the model performance by improving the image quality and tumor visibility; (ii) attention-based encoder–decoder architectures such as ARU-Net are particularly effective for multi-class brain tumor segmentation; (iii) computationally efficient models can achieve a high segmentation performance without excessive parameter overhead, which is crucial for practical deployment in clinical settings. In conclusion, the combination of pre-processing strategies and carefully designed DL architectures can substantially improve automated brain tumor segmentation, enabling more accurate diagnosis and treatment planning.

In this section, we present the experimental results obtained from the benchmark models (DenseNet121, Xception, and U-Net) and the proposed ARU-Net architecture. To comprehensively evaluate the performance, confusion matrices, multiclass ROC curves, and accuracy/loss curves were generated for each model. In addition, the quantitative performance metrics reported in Table 1 are supported by qualitative segmentation visualizations, enabling a holistic comparison across models. Table 1 summarizes the comparative performance of all models on both the original and pre-processed BTMRII datasets. As expected, the pre-processing pipeline comprising CLAHE enhancement, denoising, and Kuwahara filtering yielded significant improvements across all evaluation metrics. The proposed ARU-Net consistently outperformed the other models, achieving the highest accuracy (98.3%), F1-score (98.1%), and DSC (98.1%) on the pre-processed BTMRII dataset. Notably, ARU-Net also maintained superior performance when trained on raw unprocessed data, where it still outperformed U-Net, DenseNet121, and Xception by a considerable margin.

The observed volatility in validation loss and accuracy in Figure 10 may be attributed to the moderate batch size of eight, the complexity of the ARU-Net architecture, and the heterogeneity of the BTMRII dataset. To address this instability, the learning rate was carefully tuned to 1 × 10^−4^ with the Adam optimizer, and early stopping based on validation loss was employed. Additionally, pre-processing steps such as CLAHE, denoising, and Linear Kuwahara filtering improved the input consistency, helping to stabilize the training and enhance the generalization.

To further highlight the differences between models, segmentation outputs are visualized in Figure 11. The figure displays representative examples from each of the six tumor classes in the BTMRII dataset. For each case, the original input, Grad-CAM heatmaps, ground truth annotations, and segmentation predictions from DenseNet121, Xception, U-Net, and ARU-Net are shown. The color-coded masks illustrate class-specific tumor regions and highlight discrepancies between the model predictions and ground truth boundaries.

As illustrated in Figure 11, DenseNet121 and Xception exhibited noticeable misclassifications and boundary inconsistencies, particularly in glioma and meningioma cases. These models frequently produced incomplete or noisy segmentations, leading to reduced reliability in clinical applications. U-Net demonstrated considerably stronger segmentation capabilities, successfully capturing most tumor structures with relatively smoother boundaries. However, minor deviations, including boundary misalignments and occasional regional misclassifications, were observed in classes such as Meningioma and Other. In contrast, the proposed ARU-Net model achieved highly accurate and robust segmentation across all tumor classes. Its predictions were characterized by smooth contours, precise tumor boundaries, and minimal false classifications, even in challenging cases where other models failed. ARU-Net’s enhanced attention mechanism allowed it to adapt effectively to intra-class variability, providing superior generalization and stability. This advantage was particularly evident in complex tumor regions, where ARU-Net closely replicated the ground truth shapes without over-segmentation or under-segmentation.

Overall, these findings confirm that ARU-Net not only improves upon traditional CNN-based architectures but also extends the capabilities of U-Net in both accuracy and robustness. The integration of cross-dimensional attention and residual connections in ARU-Net enables more discriminative feature representation, contributing to its superior segmentation quality. This strong performance across both quantitative metrics and qualitative evaluations underscores ARU-Net’s potential as a reliable model for automated brain tumor segmentation in clinical practice.

As shown in Table 2, ablation experiments were conducted on the BTMRII dataset to assess the contribution of each proposed module to the overall segmentation performance. The baseline U-Net model achieved an accuracy of 93.9%, a precision of 94.3%, an IoU of 86.6%, and a DSC of 93.7%. However, it showed limitations in segmenting tumors with irregular boundaries and heterogeneous structures. After integrating the residual module (U-Net + Res), the model’s performance significantly improved across all metrics, with the DSC increasing from 93.7% to 95.6% (a relative gain of +1.9%). This demonstrates that the residual module enhances the network’s feature extraction capability and improves its ability to capture fine boundary details, thereby achieving more complete tumor segmentation. Adding the ACA module (U-Net + Res + ACA) provided a further boost, increasing the DSC to 97.0% and IoU from 91.6% to 94.3% (a gain of +2.7%), which highlights the effectiveness of ACA in refining channel attention, allowing the model to focus on the most relevant features and suppress redundant information. Despite these improvements, minor segmentation errors remained in certain fine-grained regions. Finally, by incorporating the DTA module (ARU-Net), the model achieved the best overall performance, with an accuracy of 98.3%, DSC of 98.1%, and IoU of 96.3%. These results confirm that the DTA further strengthens the model’s contextual understanding by effectively integrating multi-scale information, thereby leading to the most robust and accurate segmentation performance among all variants.

## 5. Discussion

The results demonstrate that ARU-Net provides notable advantages over traditional U-Net and other deep learning architectures. The consistent performance gains indicate that residual connections effectively mitigate gradient degradation, while ACA and DTA modules enhance multi-scale feature extraction and channel–spatial dependency modeling. These findings align with recent studies that have emphasized the role of attention mechanisms in refining segmentation accuracy [21,22,23,24,25,26,27]. Furthermore, the visual segmentation improvements observed in boundary delineation highlight ARU-Net’s potential clinical utility, as precise contours are critical for treatment planning. However, the performance variations across tumor classes also reflect the challenges posed by class imbalance, suggesting the need for future work incorporating balanced sampling or advanced augmentation strategies.

Although our framework does not include a separate feature selection or classifier stage, the impact of each architectural component was assessed through ablation studies. Specifically, residual connections, ACA, and DTA modules were incrementally added to the baseline U-Net, and their respective contributions to segmentation performance were quantified. This provides a clear understanding of how each design choice contributes to the overall effectiveness of ARU-Net.

One limitation of this study is the inherent class imbalance present in the BTMRII dataset, where certain tumor types are significantly underrepresented. To mitigate this issue, we adopted balanced mini-batch sampling and a class-weighted loss function rather than applying extensive data augmentation. This choice was made to preserve the original distributional characteristics of the dataset while still preventing bias toward the majority classes. Although effective in improving segmentation consistency across categories, future work could further investigate advanced imbalance handling strategies, such as focal loss or synthetic data generation, to enhance robustness.

While the BTMRII dataset provides high-quality manual annotations, one limitation is that intra-tumoral heterogeneity (e.g., necrosis, edema, enhancing regions) was not separately annotated. Instead, tumors were annotated as whole regions within their respective categories. This choice simplifies the segmentation task and ensures inter-rater consistency across a large dataset, but it does not fully capture the biological complexity of tumor sub-regions. Future work could leverage datasets such as BraTS, where multi-label annotations of tumor sub-structures are available, to extend ARU-Net for more fine-grained clinical applications.

Recent studies have also highlighted the effectiveness of integrating advanced deep learning strategies to enhance medical image segmentation. For instance, Sharif et al. demonstrated that hybrid convolutional recurrent architectures can significantly improve the modeling of spatial and contextual dependencies in medical images [58]. Similarly, Khalil et al. proposed an attention-guided residual network that leverages channel and spatial information to achieve more accurate delineation of tumor regions [59]. In addition, Khan et al. emphasized the importance of robust U-Net variants and demonstrated their capability to generalize effectively across diverse tumor subtypes [60]. These findings are consistent with our results, as the proposed ARU-Net also benefits from residual and attention mechanisms, confirming their value in capturing heterogeneous tumor structures and improving segmentation performance.

In comparison with recent advanced architectures, the proposed ARU-Net demonstrates distinct advantages in multi-class brain tumor segmentation. For instance, the SLCA-UNet [32] and Adaptive Cascaded Transformer U-Net [33] primarily focus on integrating transformer modules to capture global contextual information, which can improve the segmentation of large homogeneous regions but may struggle with fine-grained tumor boundaries or highly heterogeneous tumor classes. Similarly, S^3^-Mamba [34] and the Edge-interaction Mamba Network [35] leverage Mamba-based attention to enhance feature representation; however, their evaluation predominantly targets lesion sensitivity or glioma-specific datasets. In contrast, ARU-Net integrates residual connections, Adaptive Channel Attention (ACA), and Dimensional-space Triplet Attention (DTA) within a U-Net framework, explicitly designed to capture both channel-wise and spatial dependencies across multi-scale features. Experimental results on the BTMRII dataset show that ARU-Net not only achieves superior quantitative metrics (accuracy: 98.3%, DSC: 98.1%, IoU: 96.3%) compared to baseline U-Net, DenseNet121, and Xception but also provides smoother and more precise tumor boundary delineation across six heterogeneous tumor types. These findings suggest that, while transformer and Mamba-based models excel in capturing global contextual or edge-focused information, ARU-Net offers a more balanced approach by combining robust attention mechanisms with residual connections, effectively enhancing both segmentation accuracy and generalization across diverse tumor classes.

In conclusion, the experimental results demonstrate that ARU-Net delivers outstanding performance in brain tumor segmentation. Compared to the traditional U-Net and other attention-based variants, ARU-Net not only enhances the segmentation accuracy but also shows superior generalization capability. This establishes ARU-Net as an effective and practical solution for automated brain tumor image analysis, making it a strong candidate for clinical applications. Nevertheless, future work should focus on expanding evaluations to broader tumor types and larger datasets, with particular attention to addressing potential information loss in complex tumor shapes.

## 6. Conclusions

Brain tumors are among the most critical and life-threatening medical conditions, making accurate and automated segmentation of tumor regions in MRI scans essential for clinical diagnosis and treatment planning. In this study, we proposed ARU-Net, a novel DL architecture combining residual connections, ACA, and DTA modules to enhance the segmentation accuracy and robustness. The pre-processing pipeline, incorporating CLAHE, denoising, and Linear Kuwahara filtering, proved effective in improving the image quality, enhancing the feature clarity, and facilitating precise boundary delineation. Quantitative results on the BTMRII dataset show that ARU-Net significantly outperforms conventional methods, achieving 98.3% accuracy, 98.1% DSC, 96.3% IoU, and superior F1-scores, compared to DenseNet121, Xception, and U-Net. Moreover, visualizations of tumor regions demonstrate that ARU-Net provides smoother boundaries, more accurate contour delineation, and clearer differentiation of heterogeneous tumor structures than the other models. These findings confirm that the proposed architecture, combined with effective pre-processing, leads to highly reliable and precise tumor segmentation. Overall, ARU-Net presents a robust practical solution for automated brain tumor image analysis, contributing novel methodological insights to the literature and exhibiting strong potential for clinical application. Future work will focus on extending evaluations to more diverse tumor subtypes and larger datasets, while addressing challenges related to complex tumor morphology and information loss in segmentation.

## Figures and Tables

**Table 1:** Performance results of the BTMRII dataset.

Dataset	Models	Acc (%)	Pre (%)	Rec (%)	F1 (%)	IoU (%)	DSC (%)	GFlops	Param	Time/ Epoch (s)
Original	DenseNet121	77.5	82.0	75.0	78.4	65.0	78.4	412.3	8 M	45
	Xception	81.0	82.0	79.0	80.5	70.0	80.5	675.2	22.9 M	52
	U-Net	90.0	91.0	88.5	89.7	83.0	89.7	796.5	31.05 M	60
	ARU-Net	96.0	97.0	93.0	95.0	94.0	95.0	788.4	30.45 M	58
Pre-processed	DenseNet121	82.2	86.3	80.1	82.4	70.3	82.4	412.3	8 M	45
	Xception	85.3	86.7	83.3	85.2	74.3	85.2	675.2	22.9 M	52
	U-Net	93.9	94.3	93.3	93.7	86.6	93.7	796.5	31.05 M	60
	ARU-Net	98.3	99.0	95.7	98.1	96.3	98.1	788.4	30.45 M	58

**Table 2:** Ablation results of the BTMRII dataset.

Methods	Acc (%)	Pre (%)	Rec (%)	F1 (%)	IoU (%)	DSC (%)
U-Net	93.9	94.3	93.3	93.7	86.6	93.7
U-Net + Res	95.7	95.9	95.3	95.6	91.6	95.6
U-Net + Res + ACA	97.1	97.2	96.9	97.0	94.3	97.0
U-Net + Res + ACA + DTA (ARU-Net)	98.3	99.0	95.7	98.1	96.3	98.1

## Data Availability

All relevant data are fully available within the manuscript without restriction. The Kaggle data that support this study’s conclusions are publicly available online: https://www.kaggle.com/datasets/fernando2rad/brain-tumor-mri-images-17-classes (accessed on 20 August 2025).
